# Improving the Blast Resistance of Large Steel Gates—Numerical Study

**DOI:** 10.3390/ma13092121

**Published:** 2020-05-03

**Authors:** Hasan Al-Rifaie, Wojciech Sumelka

**Affiliations:** Institute of Structural Analysis, Poznan University of Technology, 60-965 Poznan, Poland; wojciech.sumelka@put.poznan.pl

**Keywords:** blast gate, steel door, shock, impact, auxetic material, damping systems

## Abstract

Blast resistant gates/doors are essential for sensitive infrastructure, such as embassies, ministries, or parliaments. Lightweight gates equipped with ‘energy absorbing systems’ have better operational performance than the traditional costly and bulky design. Graded auxetic structures have not yet been used as potential passive damping systems in the supporting frame of blast resistant gates. Consequently, this study tries to test if a uniaxial graded auxetic damper (UGAD) proposed by the authors in a recent article, namely the development of a new shock absorbing UGAD, could maintain a 3000 mm × 4500 mm steel gate operable after high blast peak reflected overpressure of 6.6 MPa, from 100 kg TNT at 5 m stand-off distance. The blast-induced response of the gate was assessed, with and without the proposed UGAD, using Abaqus/Explicit solver. Results showed that the attachment of the proposed UGAD to the gate led to a dramatic decrease in permanent deformations (a critical factor for gate operability after a blast event). Hence, a lighter, more economical gate (with 50% reduction in mass) was required to satisfy the operability condition. In addition, 49% of peak reaction forces were diminished, that have a direct impact on the supporting frame. Moreover, the results revealed that, in the numerical model, 56% of the achieved plastic dissipation energy was from the UGADs, and 44% from the gate. The outcomes of this research may have a positive impact on other sectors beyond academia, such as industry, economy, and public safety.

## 1. Introduction

Explosive attacks on civilian structures have recently increased [1,2], requiring more robust protecting systems. Traditionally, blast-resistant doors rely on strength and mass to provide protection from explosions. The dynamic response of steel or steel-concrete blast doors have been covered in research [3] and in engineering standards [4]. For instance, the United Facilities Criteria (UFC) [4], provides the engineering design steps for blast resistant doors with two illustrative examples. The first example is a double-leaf built-up A36 steel door with dimensions 6 × 8 ft. (1830 mm × 2438 mm). The door has to sustain low blast pressure of 14.8 psi (0.1 MPa) and leakage is permitted. A $\frac{3}{4}$ inch plate (19 mm thick) with L 4 × 3 × $\frac{1}{2}$ satisfied the requirements. The second example is a single-leaf steel door with dimensions 4 × 7 ft. (1219 mm × 2133 mm). The door has to sustain high blast pressure of 1100 psi (7.5 MPa) and leakage is not permitted. A two-inch plate thickness (50.8 mm) was required to satisfy the design. However, from an operational or economic point of view, these massive doors are not suitable for general-purpose usage, as they are too heavy [5,6]. Current needs require a blast door to be lightweight and blast protective [7,8].

The addition of stiffeners to blast doors has been investigated by several researchers such as Hsieh et al. [9], Mohammed et al. [10], Goel et al. [11], and Veeredhi and Rao [12]. For example, Hsieh et al. [9] analysed the performance of a blast door with different dimensions of an I-shaped inter stiffener. The door consists of a rectangular steel plate measuring 5410 mm × 2560 mm × 20 mm. The I-shaped stiffener width is 120 mm which has been kept constant, while the depth and web thickness were optimized through the study. The ratio of stiffener’s stress to plate stress was the key factor to evaluate the influence of the stiffener. As a result, the door was capable of sustaining a localized pressure of 2.5 MPa, which is more than the recommended value by TM5-1300 technical manual, [13].

Recent advances in blast resistant doors suggest hierarchical stiffened door structures [5,14,15]. Hierarchical topology leads to a lighter [16], and more stiff structure in global deformation [17,18]. Meng et al. [5] propose a new blast resistant door structure with a hierarchical stiffened structure using sheet molding compound (SMC) material reinforced by carbon fiber reinforced plastic (CFRP). The door (dimensions 900 mm × 1800 mm, weight = 120 kg) was designed, manufactured, and tested (experimentally and numerically). Results show that the composite door structure is strong and stiff enough to resist a peak pressure of 0.45 MPa from a blast wave [5]. Orthogrid stiffened structures are also used as easily made anti-impact solution [19,20,21]. The explosion experiments of Zhao et al. [22], revealed that an orthogrid stiffened SMC door structure can resist 0.4 MPa blast overpressure, showing elastic response as a basic protection requirement.

Arched panels with arc shape are developing blast protective solutions [23,24]. The blast resistance properties of arched blast doors were investigated [25,26] using one arch that transfers the blast load to the arch supports. However, this technique may, in return, require very strong supports. Chen and Hao [27] introduce a new configuration that consists of a double-layered panel with a structural form of multi-arched-surface. Blast resistance and energy absorption capacities were numerically investigated using FE code. Using parametric studies to find the optimum design, the research proved that multi-arch panel performs better than other forms of panel, i.e., can sustain higher blast loads [27]. One of the studies recommends the use of accordion-flex door [28]. The proposed door is an accordion panel that is allowed to deform significantly when exposed to blast pressure. The lightweight door showed to withstand 50 psi (0.34 MPa) peak reflected overpressure.

Patented technical solutions are also available such as “lightweight armoured panels and doors” [29] and the “ablative blast resistant security door panel” [30]. Manufacturers usually make use of the patented ideas and standards to construct those blast resistant doors. A Korean company, namely SH Door Tech Co., produces a 11.3 m large gate that is supposed to resist 50 bars (5 MPa) of blast pressure. A Finnish company, TEMET, produces the so-called “SO-6 double wing blast door”. The door is fabricated from a steel plate stiffened by I-beams. Different sizes are available with a maximum possible width of 4900 mm and height of 4000 mm leading to 20 tons of weight (1 ton/m^2^). The manufacturer confirms that the door has resistance against multiple blast loads ranging from 9–18 bars (0.9–1.8 MPa) peak reflected overpressure, and that the steel material behaves within the elastic range.

It is evident from the reviewed blast resistant gates, that the design of a relatively light-weight gate that could sustain 6.6 MPa high intensity peak reflected overpressure is a challenging target in this study. First, the structural response of a 3000 mm × 4500 mm steel gate is numerically assessed, without any supplementary damping systems. The results are then compared with the performance of the gate equipped with uniaxial graded auxetic dampers (UGAD). The UGAD was recently proposed by the authors in an article, namely the development of a new shock absorbing UGAD [31]. It is important to highlight here that the numerical parameters for the steel material used in this study are based on the in depth experimental tests of Børvik et al. [32], where detailed material constants are evaluated for a coupled computational model of visco-plasticity and damage. The research of Børvik et al. [32] showed that their proposed constitutive model and material constants, were efficient in predicting deformation and fracture in ductile materials during penetration and impact.

## 2. Site Plan and Assumptions

**Figure 1:**
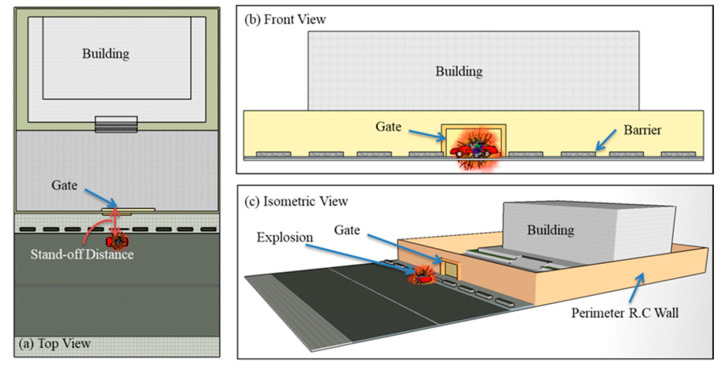
Top (**a**), front (**b**) and isometric (**c**) views of the blast scene.

The gate is assumed to secure the main entrance of a critical structure, such as an embassy, ministry, or hospital, that needs the highest level of protection, Grade IV (refer to Dusenberry [33], buildings levels of protection). In other words, no evacuation is needed, and only superficial damage is expected. The building is surrounded by a reinforced concrete perimeter wall of height 4 m. The gate is supported by that wall and works as the main access to the premises. Figure 1 shows the explosion source, stand-off distance, gate under consideration, reinforced concrete perimeter wall, and building.

In terms of the gate itself, the blast performance of gate assembly supposed to be in Category II of ASTM F2247-03; where the gate stays operable with small permanent deformation. According to UFC [4], an ‘operable’ door after a blast event can be achieved when door edge rotations do not exceed 2°. In addition, the following assumptions were made in this study:

- The gate is outside the explosion fireball. In other words, the interaction with the produced gases can be neglected and there is no afterburning effect. Afterburning (combustion of the detonation products following an explosion) can increase the duration of the positive phase and thus the impulse on near field structure.
- As a blast wave propagates in the air, atmospheric pressure is an important factor which varies with the altitude of the location. Therefore, it is assumed here that the blast occurs at sea level.
- The charge was uncased with no additional loading from fragmentation (for more information about fragmentation, refer to Szymczyk et al. [34].

## 3. Geometrical and Material Properties of the Gate

The entrance, where the blast resistant gate supposed to be attached, is required to have 2600 mm clear height and 4100 mm clear width. These dimensions are appropriate for the entry of small to medium-size vehicles in addition to a pedestrian lane on one side. The total dimensions of the sliding steel gate are 3000 mm high and 4500 mm wide (providing 200 mm of bearing surface on the supports and satisfying the clear opening requirement mentioned previously). Based on the results of research done by the authors [35], the case when two opposite sides of a gate are simply-supported, and the other two are free, abbreviated as “SFSF” case, was selected as the best boundary condition. Hence, the gate was assumed here to be sliding between two gutters, i.e., the longest horizontal sides are simply supported, and the shortest vertical sides are free (working as a one-way slab).

**Figure 2:**
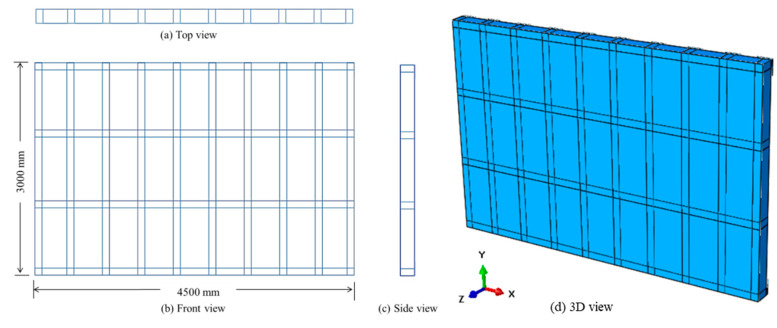
Top (**a**), front (**b**), side (**c**) and 3D view (**d**) of the steel gate.

The structure of the gate consists of a steel frame welded to front and back steel plates. The frame consists of 10 vertical and 4 horizontal rectangular steel hollow sections of 180 mm × 100 mm with c/c spacing of 500 and 1000 mm, respectively. The use of more vertical steel hollow sections (with less spacing) is related to the fact that the gate works as a one-way slab supported at the top and bottom edges. Front, top, side and 3D views of the gate are shown in Figure 2. The rectangular hollow sections assumed to have the same thickness t as the front and back plates. The gate dimensions are constants while the thickness t is variable. Four different values of t were considered, which are 2.5, 5, 7.5, and 10 mm, giving four different gates, abbreviated here as, G2.5, G5, G7.5 and G10, respectively.

Weldox 460E steel material has been used for both the plates and the hollow sections due to its high strength and ductility. More details on the material model of Weldox 460E Steel will be presented in Section 5, where Table 1 provides detailed material parameters.

## 4. Threat Assessment and Blast Loading

Terrorist attacks are made by transporting explosive materials near the target point, where the mass of the explosives (M) and stand-off distance (R) are the main two factors to assess. The US Department of Homeland Security, in their reference manual [36], provide a range for the mass, in TNT equivalency. The range is based on the method of transport, which might be through a luggage, automobile, vans or even truck. The maximum possible carried mass is defined as 100 lb (45 kg) for a luggage, 450 lb (204 kg) for a normal sedan car, 4000 lb (1814 kg) for a van, and 100,000 lb. (45,359 kg) for a truck. As the sensitive building, described in Section 2, is situated in an urban area, trucks should be excluded and prevented from approaching the area. The remaining possibilities are either luggage (45 kg), car (~200 kg) or van (~2000 kg).

To satisfy the assumptions made in Section 2, a stand-off distance greater than the target longest dimension, prevents near-field effect and provides uniform blast pressure. Yuen et al. [37] state that “when the stand-off distance exceeds the largest plate dimension, loading could be considered to be uniform”. In the current case, the longest side of the gate is 4500 mm, is the minimum stand-off distance that can satisfy assumptions made in Section 2. The greater the stand-off distance, the less peak reflected overpressure would be on the gate, for a specific TNT mass and incident angle. Barriers are the usual used mechanisms to achieve a certain stand-off distance. According to literature survey of current blast resistant doors in Section 1, it was concluded that the design of a relatively light-weight, 3000 mm × 4500 mm gate that could sustain 6.6 MPa of pressure is a challenging target in this paper. Therefore, the 6.6 MPa is set as the criteria required to be met rather than a specific mass or stand-off distance. However, to represent the 6.6 MPa target, a combination of 100 kg of TNT at 5 m was chosen here as ConWep input parameters. ConWep is a conventional weapons effects calculation tool in Abaqus. This peak reflected overpressure can also be achieved from other M-R combinations, such as 45 kg luggage at 3.8 m, 200 kg car at 6.2 m or 2000 kg van at 13.5 m. All M-R combinations above have the same scaled distance $Z=\frac{5\;m}{\sqrt[{3}]{100\;\mathrm{kg}}}=$ 1.07 $m/{\mathrm{kg}}^{1/3}$, which is more than the minimum scaled distance 0.4 $m/{\mathrm{kg}}^{1/3}$ required to avoid close-range detonations [38,39].

**Figure 3:**
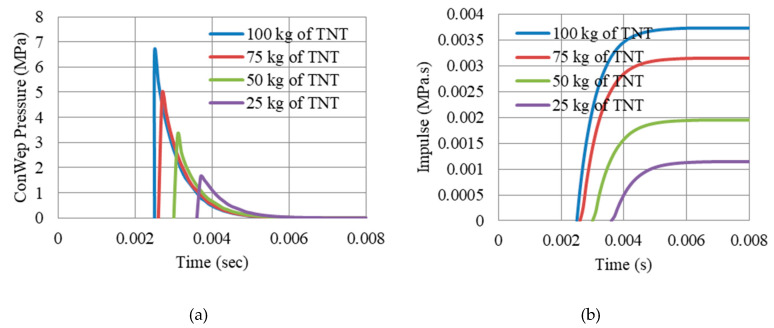
Peak reflected overpressure and impulse history of 4 blast levels (25 kg, 50 kg, 75 kg and 100 kg, R = 5 m), (**a**) peak reflected overpressure, (**b**) impulse.

The gates, G2.5, G5, G7.5, and G10, are assessed against 4 levels of blast pressures, 1.65 MPa, 3.3 MPa, 4.95 MPa and the maximum 6.6 MPa, achieved from 25, 50, 75, and 100 kg of TNT at R = 5 m, respectively. The levels are useful to evaluate the corresponding variation in reaction forces (Section 7) and the influence of the passive damping systems (Section 8) for each specific level. The peak reflected overpressure time history of the 4 levels is shown in Figure 3, based on ConWep loading on the gate frontal plate.

**Figure 4:**
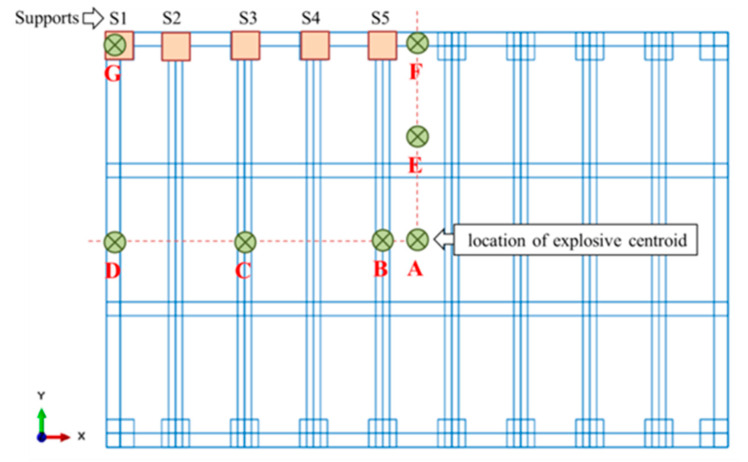
Schematic of explosive centroid effective locations, situated on the gate projection, M = 100 kg, R = 5 m.

While the stand-off distance R was set as 5 m, the centroid location of the explosive mass may be located anywhere on a plane parallel and 5 m apart from the gate. However, as known, peak reflected overpressure occurs when the angle of incident is 0° (the angle between outward normal of the target and the direct vector from explosive charge toward that point). In other words, points located outside the gate projection have less effect, and are hence excluded. In addition, while the gate (and its supports) is symmetric, possible positions can be taken on quarter of the gate and their effect on nearest supports can then be evaluated. Figure 4 shows 5 highlighted supports S1–S5, and 7 different positions of explosive centroid, denoted as A–G. Results showed that taking different possibilities of explosive positions located on a gate projection with R of 5m had a minor effect on reaction forces (abbreviated hereafter as RFs, or RF for single reaction force) of up to 10%. Hence, the possibilities were omitted and the centre of the gate was selected as the default location.

## 5. Numerical Modelling

FE codes and numerical simulations are cost-effective tools in the field of blast protective design [40,41]. Therefore, the problem under investigation was numerically modelled using Abaqus/CAE and analysed using Abaqus/Explicit solver. The gate (frame and plates) where modelled using 3D deformable shell parts with 5 points of integration along the thickness t. A homogenious isotropic steel section was defined.

Material behaviour was modelled as elasto-plastic with Johnson-Cook (J-C) strain hardening and damage initiation, which can occur due to different blast pressure intensities. Johnson-Cook material model is one of the semi-empirical constitutive models that can describe the plastic material behaviour at high strains, high strain rates and high temperatures. The model (in Equation (1)) describes the yield stress $\sigma_{y}$ and takes into account the strain rate hardening and thermal softening effects [42,43,44,45]. The dimensionless temperature parameter $\hat{T}$ is defined in Equation (2).

(1) $$\sigma_{y}=\left(A+B\;\varepsilon^{n}\right)\;\left[1+C\;\ln\left(\frac{\dot{\varepsilon }}{\dot{\varepsilon_{0}}}\right)\right]\;\left[1-{\left(\hat{T}\right)}^{m}\right]$$

(2) $$\{\begin{array}{c} \hat{T}=0\;\mathrm{for}\;T<T_{0}\\ \hat{T}=\frac{T-T_{0}}{T_{m}-T_{0}}\;\mathrm{for}\;T_{0}<T<T_{m}\\ \hat{T}=1\;\mathrm{for}\;T>T_{m} \end{array}$$

where ε is the plastic strain, $\dot{\varepsilon }$ is the plastic strain rate, $\dot{\varepsilon_{0}}$ is the reference plastic strain rate, *T* is the current material temperature, $T_{m}$ is the melting point of the material, and $T_{0}$ is the transition/room temperature at or below which there is no temperature dependance of the yield stress. *A*, *B*, *C*, *n* and *m* are material parameters measured at or below $T_{0}$. *A* is the yield stress, *B* is the pre-exponential factor, *C* is the strain rate factor, *n* is the work-hardening exponent, and *m* is the thermal-softening exponent. 

In addition, the Johnson–Cook dynamic failure model is supplied by Abaqus/Explicit [46]. The failure is assumed to happen when the damage parameter ω exceeds 1. The damage parameter is defined as:

(3) $$\omega =\sum \left(\frac{\Delta \varepsilon }{\varepsilon_{f}}\right)$$

where Δε is an increment of the plastic strain, $\varepsilon_{f}$ is the plastic strain at failure, and the summation is performed over all increments in the analysis. The plastic strain at failure $\varepsilon_{f}$ is dependent on the nondimensional plastic strain rate $\frac{\dot{\varepsilon }}{\dot{\varepsilon_{0}}}$, pressure to *HMH* stress ratio $\frac{p}{q}$, and the dimensionless temperature parameter $\hat{T}$, where *HMH* is Huber–Mises–Hencky criterion (known as von Mises yield criterion). The strain at failure $\varepsilon_{f}$ can be expressed as:

(4) $$\varepsilon_{f}=\left[d_{1}+d_{2}\exp\left(d_{3}\;\frac{p}{q}\right)\right]\left[1+d_{4}\;\ln\left(\frac{\dot{\varepsilon }}{\dot{\varepsilon_{0}}}\right)\right]\;\left(1+d_{5}\;\hat{T}\right)$$

where $d_{1}-d_{5}$ are failure parameters. All material parameters for J–C model are presented in Table 1. They are based on the detailed experimental tests of Børvik et al. [32] for Weldox 460E Steel, that include not only mechanical properties, but also the chemical composition of that type of steel.

The 14 rectangular sections of the frame and the 2 plates were assembled so that the length of the gate is parallel to x-axis, the height to y-axis while blast pressure and corresponding deflections follow z-axis, Figure 2d. A non-linear dynamic explicit step was used with total time of 0.02 s. The “Adiabatic heating effects” were also added to include the effect of heat generated from plastic strains, setting the inelastic heat fraction χ=0.9.

To represent the welding, the 16 parts were connected using “Tie” constraint. An explicit “general contact” was also defined for the whole model, with tangential and normal behaviour contact property options. For the tangential behaviour, a “penalty” friction formulation was selected with coefficient of friction = 0.3. For the normal behaviour, “hard” contact was chosen. As mentioned earlier in Section 4, to model the blast loading, ConWep was used. As a blast targeting the gate is expected to be near the ground, “surface blast” was chosen rather than the “air blast” option. This is basically to account for reflections from the ground surface; which in return; would produce more peak reflected overpressure on the gate than the “air blast” option.

**Figure 5:**
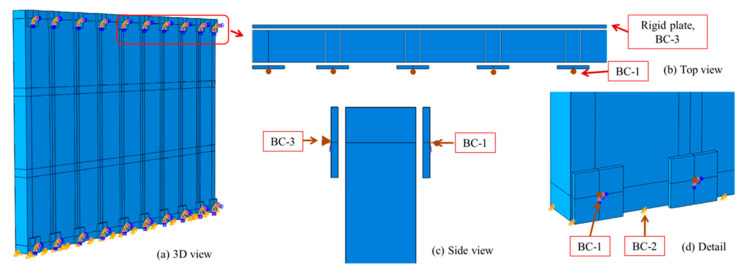
Applied boundary conditions (**a**) 3D view, (**b**) top view, (**c**) side view and (**d**) detail.

In terms of boundary conditions (BC), and to replicate the behaviour of the gate in reality, three BC were defined (Figure 5). At time of positive blast pressure, 20 separated square steel plates of 200 mm × 200 mm × 10 mm were placed behind the gate, on top and bottom sides, to hold the gate. The plates are 10 mm apart from the gate and coincident with the centre line of frame sections. The 1st boundary condition, BC1 is specified at the centre of those plates as ‘Pin’ constraining only translational DOF, as shown in Figure 5a,d. BC1 will provide nodal reaction forces that would be easier to interpret and compare. The gate is assumed to be sliding on rollers that would allow it to move in the x-z plane with limited movement in y direction. This is presented by BC2, which limits the movement of the gate in y-axis at initial step. BC2 is applied at bottom edges of the gate itself. At time of negative blast pressure, the gate is held from re-bound action by 2 long rigid steel plates (200 mm × 4500 mm × 10 mm), one at the top and one at the bottom, with 10 mm gap from the gate, as shown in Figure 5b,c. BC3 is applied at the centroid of those plates to restrict translational and rotational degrees of freedom.

**Figure 6:**
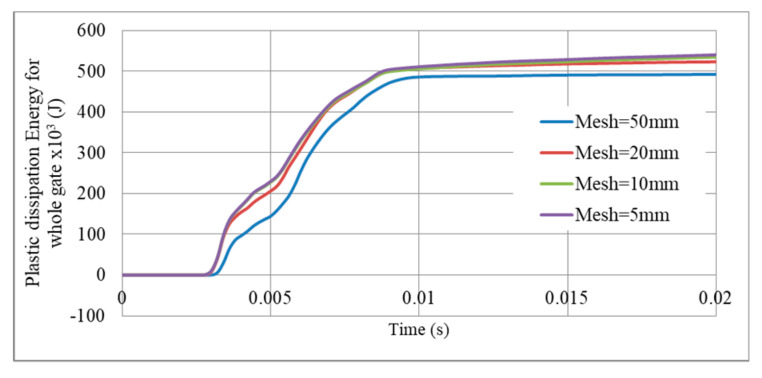
Variation of plastic dissipation energy with mesh size, Gate G5, M = 100 kg TNT, R = 5 m.

**Figure 7:**
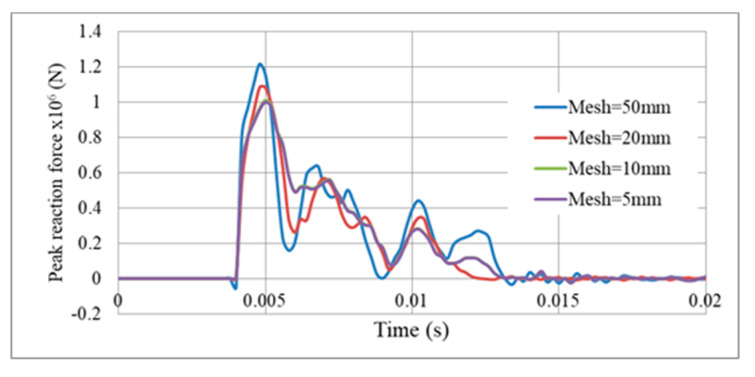
Reaction force variation with mesh size, Gate G5, support S5, M = 100 kg TNT, R = 5 m.

The mesh consists of linear S4R element, which is a 4-node doubly curved shell with reduced integration. As known, computational cost is a key factor in numerical simulations and the “less expensive—more accurate” model should be selected at early stages [47,48]. Therefore, a detailed analysis of mesh size was conducted to validate the numerical model based on plastic dissipation energy of the gate and peak reaction force at middle supports. Mesh size (or finite element size) of the gate G5, as an example, was varied from 5, 10, 20, and 50 mm. Results revealed that plastic dissipation energy and reaction force for mesh size 5 mm and 10 mm are nearly coincident (<1% error), as shown in Figure 6, Figure 7 and in Table 2. However, results for mesh size 20 mm slightly deviated with more error perceived in the 50 mm option (Table 2). So, the 10 mm mesh size was selected for future simulations of the gate, as it is the less expensive and more accurate model.

## 6. Uniaxial Graded Auxetic Damper (UGAD)

**Figure 8:**
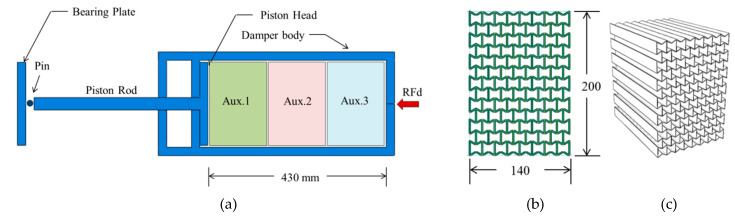
Uniaxial Graded Auxetic Damper (UGAD). (**a**) Components of the UGAD. (**b**) Cross-section. (**c**) 3D.

The parametric study presented by Al-Rifaie and Sumelka [31] focused on six parameters that had to be optimized for better performance of the UGAD. The selected parameters were loading direction, cell dimension, aluminium grade, cell angle θ, effective number of layers, and lastly, cell wall thickness t. For more details on the numerical modelling, parametric study and detailed properties of the UGAD, refer to Al-Rifaie and Sumelka [31]. Figure 8 shows the components of the UGAD, with the cross-section and 3D view of one auxetic core.

The final geometrical and mechanical properties of the three auxetic cores are described in Table 3. They have the same *L*, θ, material grade, size, and hence, overall volume. The cell-wall thickness t is the variable parameter which, in return, leads to distinct impact absorption potentials.

**Figure 9:**
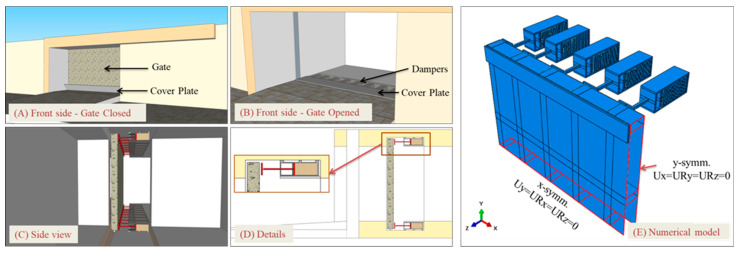
The proposed gate system in reality, and the numerical modeling of quarter the system (due to symmetry).

The steel gate, UGADs and virtual supporting concrete structure are shown in Figure 9. It presents where those sacrificial auxetic cores are situated in relation to the whole system. According to Figure 4, the gate requires twenty UGADs uniformly distributed (10 at the top and 10 at the bottom, Figure 9C) to absorb potential blast energy. However, due to symmetry, the numerical modelling of quarter the system would be sufficient (Figure 9E).

## 7. Gate Response (without UGAD)

### 7.1. Peak Nodal Reaction Forces

In this section, the nodal reaction forces at supports S1–S5 (Figure 4) were quantified and the effect of blast levels and gate mass were studied. As known, the pin support (BC1 in Figure 5) provides 3 components of reaction forces, RFx, RFy and RFz. However, simulations showed that RFx and RFy are very small compared to RFz. Therefore, RFz is the considered component in this study and hereafter denoted as RF as it is the prominent one.

**Figure 10:**
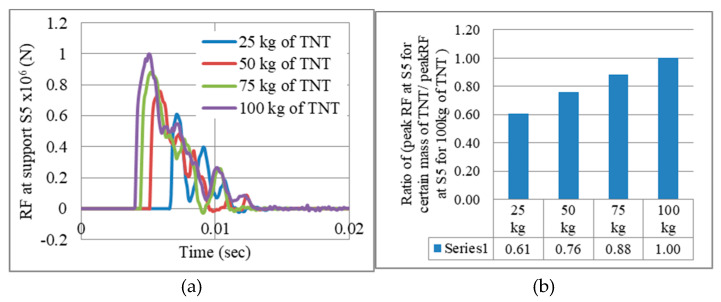
Effect of blast levels (changing mass of TNT) on peak nodal reaction forces, RFs. (**a**) RF time history. (**b**) RF_m_/RF_100_.

As mentioned in Section 4, the gate is assessed against four blast levels of peak reflected overpressures, 1.65 MPa, 3.3 MPa, 4.95 MPa, and the maximum 6.6 MPa, achieved from 25 kg, 50 kg, 75 kg, and 100 kg of TNT at R = 5 m, respectively. It is obvious that a passive damper designed for 100 kg of TNT would be too stiff if a blast of 50 kg of TNT occurs. Hence, a “graded” auxetic system (Section 6) was suggested to absorb reaction forces resulting from different blast levels (TNT mass). Figure 10a shows the reaction force-time history at support S5 for different masses of TNT. The ratio of peak RF for certain mass of TNT to the peak RF for 100 kg of TNT (RF_m_/RF_100_) is presented in Figure 10b. Results show that the quarterly-decreasing mass of TNT did not reduce peak RF in the same pattern. For instance, a reduction from 100 kg to 50 kg in the mass of TNT led to only 24% fall in the peak RF at the same support (RF_m_/RF_100_ = 76%). In other words, the blast level–reaction force relation is not proportional. Hence, the performance of passive dampers should be analysed for each blast level separately.

**Figure 11:**
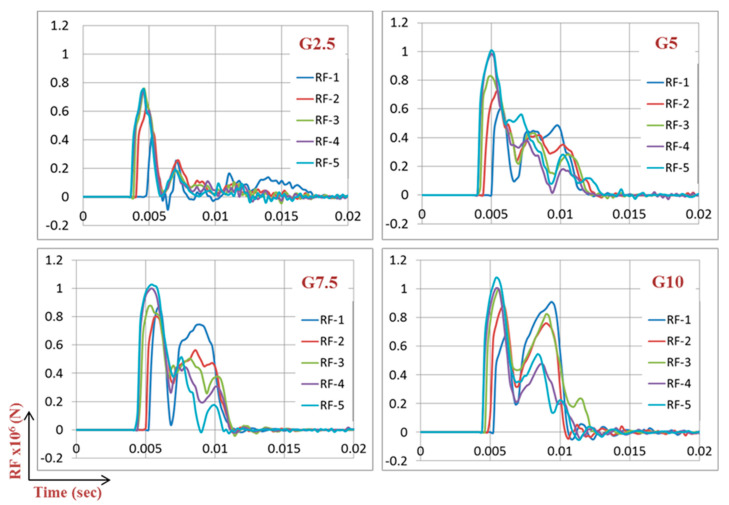
Reaction forces for the 4 gates G2.5, G5, G7.5 and G10, under a blast of 100 kg of TNT, R = 5 m.

The effect of the mass of the gate on RFs should also be evaluated. The mass of the four gates are shown in Table 4 ranging from 1.1 ton for the G2.5 to 4.38 tons for G10. Results (Figure 11) showed that when mass was increasing, corresponding reaction forces were slightly increasing except the initial sharp rise of 23% in peak RF between G2.5 and G5. For instance, doubling the mass from 2 to 4 tons (gates G5 to G10) led to slight increase in peak RFs of only 7%. Broadly, the selection from G5, G7.5 or G10, would have slight effect on RFs and hence, the same designed UGAD (Section 6) may work for all of them. Moreover, a second peak in the reaction forces can be noticed (Figure 11), which becomes more and more prominent as the gate thickness increases. This can be justified due to the increase in the mass, stiffness, and hence, rebound effect of the gates.

### 7.2. Deformation and Operability Analysis

**Figure 12:**
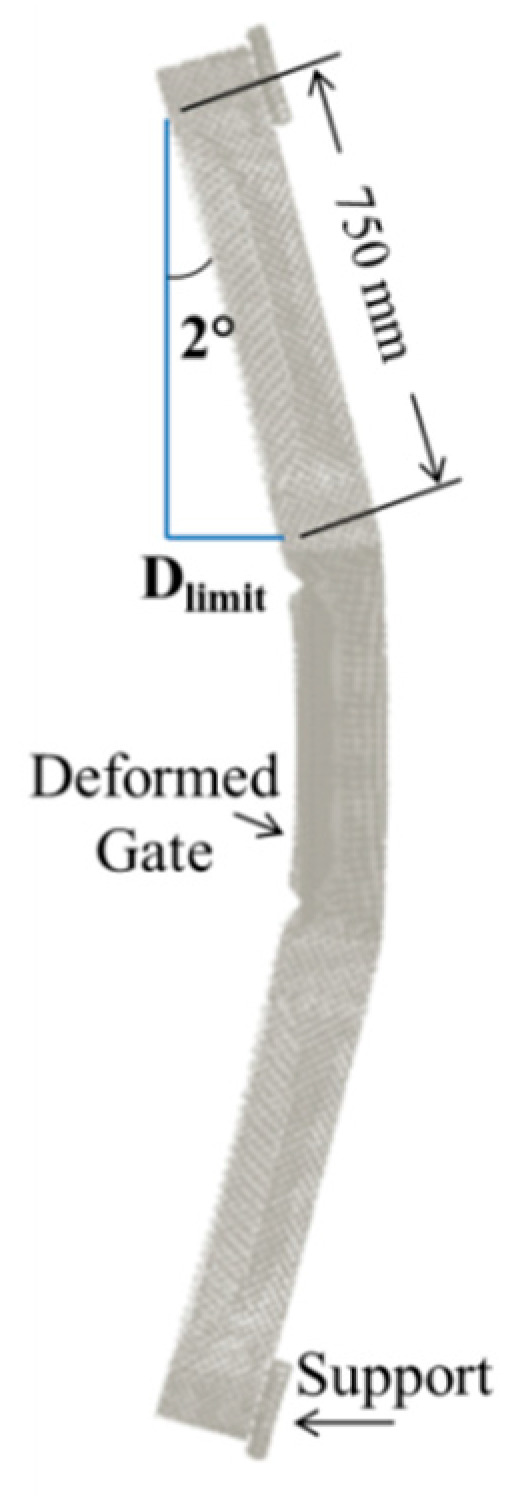
Deformation limit.

**Figure 13:**
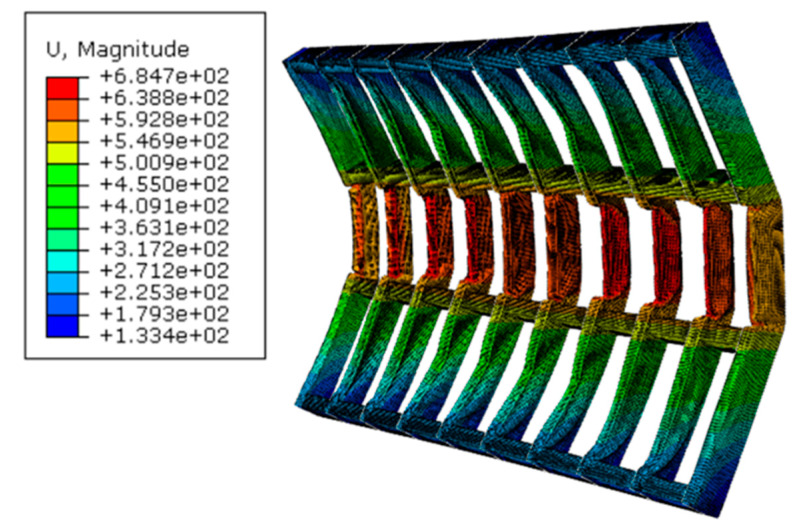
Displacement of G2.5 (6.6 MPa peak reflected overpressure).

The performance of the 4 gates, G2.5, G5, G7.5, and G10, were assessed based on maximum plastic strain, permanent deformation and corresponding operability. The behaviour was addressed for the peak reflected overpressure 6.6 MPa (from 100 kg of TNT at R = 5 m). As mentioned in Section 2, according to UFC [4], an ‘operable’ door after a blast event can be achieved when door edge rotations do not exceed 2°. The primary supporting elements in the gate are the vertical rectangular hollow sections. Their deformation affects operability after a blast event. As shown in Figure 12, a 2° rotation of an unsupported length of 750 mm leads to a deformation limit D_limit_ = 750 sin2° = 26.2 mm. If permanent deformation exceeds that limit, then the gate can be considered as inoperable. As an example, Figure 13 shows the catastrophic failure of gate G2.5.

The detailed results for peak plastic strain (PEEQ) and permanent deformation (*d*) are listed in Table 5, for the frame, front plate and back plate. The term “plastic strain” means the “maximum equivalent plastic strain through plate thickness integration points” while the “peak” considers taking the extreme value in gate component (e.g., frame). Results show that PEEQ and d values were decreasing dramatically with increasing the thickness t. In addition, *d* values for G2.5, G5 and G7.5 were more than 26.2 mm (D_limit_). In other words, G10 was the only gate that can be considered as operable after the blast event, with permanent frame deformation *d*_frame_ = 4.4 mm. The addition of passive dampers in Section 8 may reduce d values for G5 or G7.5 to D_limit_, i.e., a lighter and hence more economical gate may be used (which is one objective of this study).

**Figure 14:**
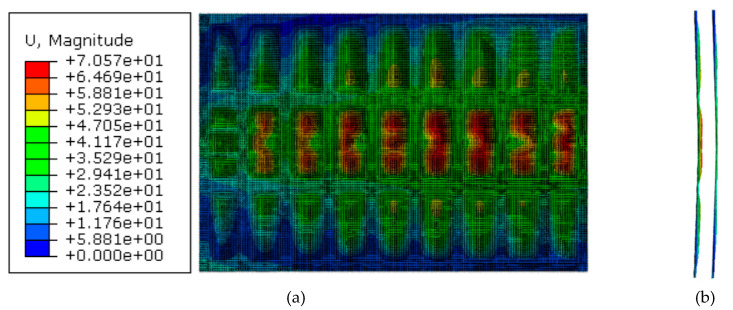
Spatial displacement of front and back plates of gate G5 after 6.6 MPa peak reflected overpressure. (**a**) Front view. (**b**) Side view.

The response of the front and back plates should also be analysed as their excessive deformation could cause integrity problems for the frame in addition to an undesired aesthetic for the whole gate. As the front and back plates were welded to the frame, no damage initiation was noticed. Welded areas were moving consistently with the frame, while the deformation of unsupported areas was as large as 40 mm for G2.5, and as small as 6 mm for G10. Figure 14 show the spatial displacement of front and back plates of gate G5 after 6.6 MPa peak reflected overpressure.

**Figure 15:**
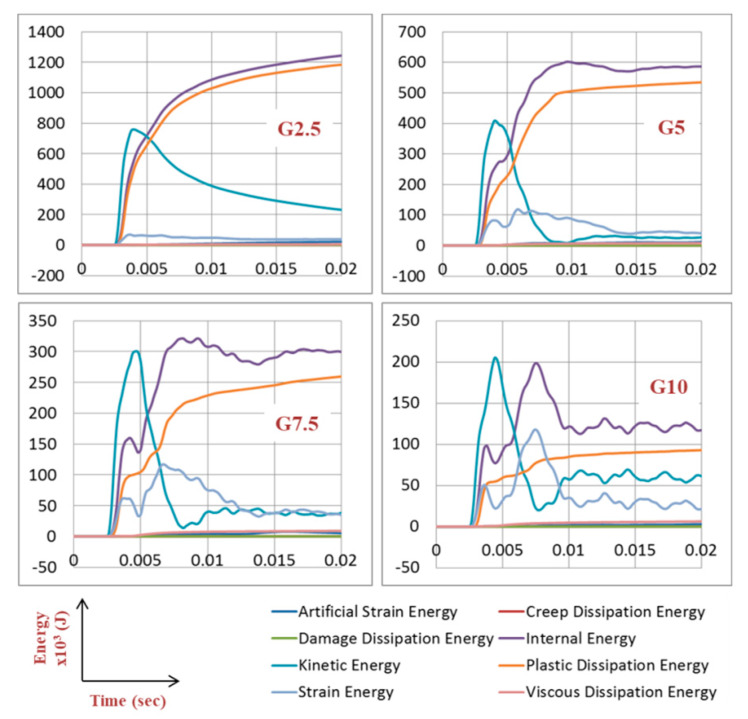
Energy components for the 4 gates G2.5, G5, G7.5 and G10, under blast of 6.6 MPa (from 100 kg of TNT, R = 5 m).

As the aim of this paper is the design of a blast resistant gate supported with passive dampers to absorb more blast energy, it is critical at this stage to understand the energy dissipation of the gate itself. An explosion of 100 kg of TNT releases 461.2 × 10^6^ J of energy at the position of detonation. However, the gate receives much less energy depending on stand-off distance and exposed area of the gate. Figure 15 shows energy components for the four gates, namely G2.5, G5, G7.5, and G10, under a blast of 6.6 MPa (from 100 kg of TNT, R = 5 m, explosive location A). The following points can be highlighted; The more is the mass of the gate, the less the kinetic energy is (e.g., peak kinetic energy for G2.5 is 4 times higher than G10). Plastic dissipation energy and strain energy are the main components of internal energy in the gate. The plastic dissipation energy is found to be decreasing with increasing the thickness t. This is linked to the plastic deformations that are normally less for higher values of t. The plastic dissipation energy was as high as 1200 × 10^3^ J for G2.5, and as low as 90 × 10^3^ J for G10. In other words, light gates provide better energy absorption at the cost of more permanent deformation. Strain energy found to be increasing with increasing the thickness t. Damage dissipation energy was zero as damage criteria were not met. Viscous and creep dissipation energies were also zero. Artificial strain energy was very small (up to 2% of the total internal energy), which reflects the accuracy of the numerical model.

## 8. Gate Behavior with the Proposed Auxetic Damper

The achievement of a lighter and hence more economical gate is one of the objectives of this study. In Section 7, the performance of the four gates G2.5, G5, G7.5 and G10 (on rigid supports) were assessed, based on maximum plastic strain, permanent deformation, and corresponding operability. The behaviour was addressed for the peak reflected overpressure of 6.6 MPa (from 100 kg of TNT at R = 5 m). Gate G10 was the only gate that can be considered as operable after the blast event, with peak *d*_frame_ = 4.4 mm, less than D_limit_ (26.2 mm).

In this section, the behavior of the remaining three gates, G2.5, G5 and G7.5, was assessed with the application of the proposed uniaxial graded auxetic damper (UGAD) designed earlier. Table 6 shows plastic strain, permanent deformation and operability of the gates with the proposed auxetic damper, subjected to 6.6 MPa blast pressure from 100 kg TNT at R = 5 m. Both G7.5 and G5 were passed the operability requirement with *d*_frame_ < D_limit_ (26.2 mm). The frame permanent deformation of G7.5 dropped from 28.4 to 4mm with the addition of the UGADs. Furthermore, the frame permanent deformation of G5 decreased from 40.5 to 22 mm with the addition of the UGADs, making G5 the lightest-operable option that can withstand the peak reflected overpressure target of 6.6 MPa.

**Figure 16:**
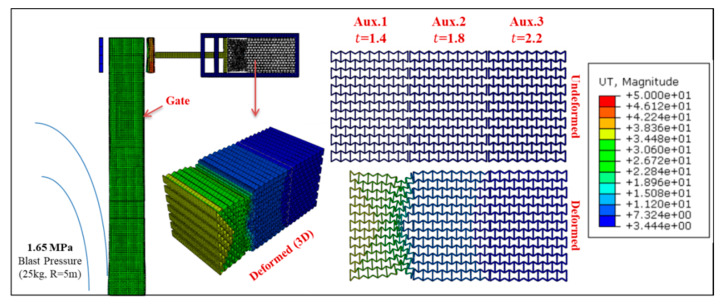
Displacement of Gate G5 and the Auxetic damper after a blast peak reflected overpressure of 1.65 MPa from 25 kg TNT at R = 5 m.

**Figure 17:**
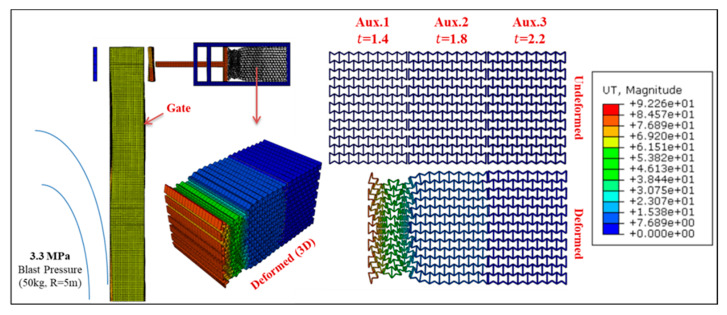
Displacement of Gate G5 and the Auxetic damper after a blast peak reflected overpressure of 3.3 MPa from 50 kg TNT at R = 5 m.

**Figure 18:**
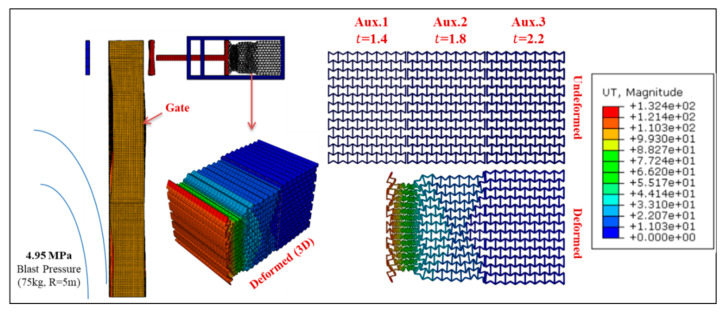
Displacement of Gate G5 and the Auxetic damper after a blast peak reflected overpressure of 4.95 MPa from 75 kg TNT at R = 5 m.

**Figure 19:**
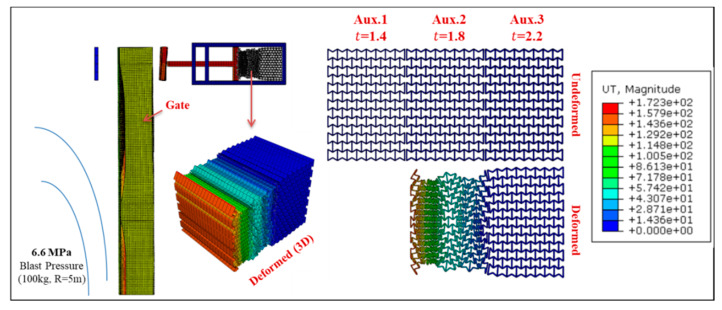
Displacement of Gate G5 and the Auxetic damper after a blast peak reflected overpressure of 6.6 MPa from 100 kg TNT at R = 5 m.

Permanent deformation of Gate G5 and the UGAD (at support S5, Figure 4) are shown in Figure 16, Figure 17, Figure 18 and Figure 19, for different blast pressures. It is explicit that up to 3.3 MPa blast pressure (Figure 16 and Figure 17), the 1st auxetic core (Aux.1) was the only deformed one with maximum deformation of 92 mm. In other words, only Aux.1 has to be changed after such a blast event. Nonetheless, blast pressures between 3.3 and 6.6 MPa (Figure 18 and Figure 19), induces a plastic deformation in both Aux.1 and Aux.2 cores, i.e., both of them should be replaced after such a high blast event. Although Aux.3 (presented in Section 6) was supposed to absorb the 6.6 MPa blast pressure, the first two cores were able to absorb the impact up to their capacity without deforming the third core (Aux.3). This is an extremely important advantage. Hence, Aux.3 hereafter will work as a factor of safety for unexpected higher blast loads or multiple explosions in a short period of time.

**Figure 20:**
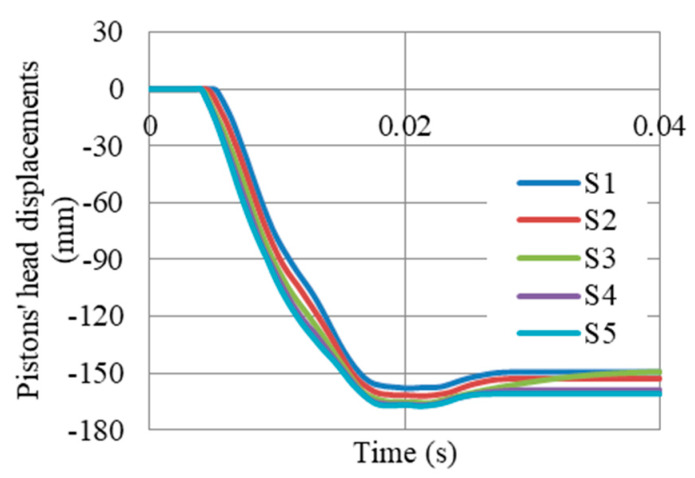
Displacements of Pistons’ heads (i.e., compressed length of auxetic cores) at supports S1–S5, after peak reflected overpressure of 6.6 MPa from 100 kg TNT at R = 5 m, Gate G5.

**Figure 21:**
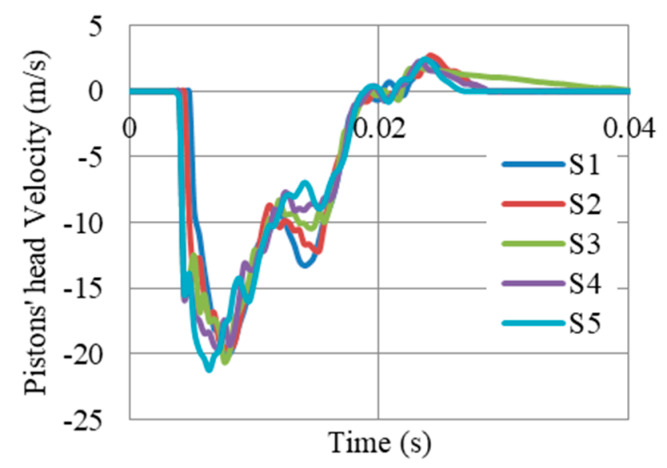
Velocity of Pistons’ heads (i.e., velocity of compressing auxetic cores) at supports S1–S5, after a peak reflected overpressure of 6.6 MPa from 100 kg TNT at R = 5 m, Gate G5.

The displacements of pistons’ head (i.e., compressed length of auxetic cores) at supports S1–S5 (Figure 20), shows the integrity of the gate and the movement as one large body. The maximum was 167 mm at S5, while the lowest was 161 at S1. So, the difference was only 6 mm. The results can also be presented in terms of pistons’ head velocity (i.e., velocity of compressing auxetic cores). Figure 21 shows that the velocities of compressing auxetic cores in all UGADs were coincident, with peak velocity of about 20 m/s.

**Figure 22:**
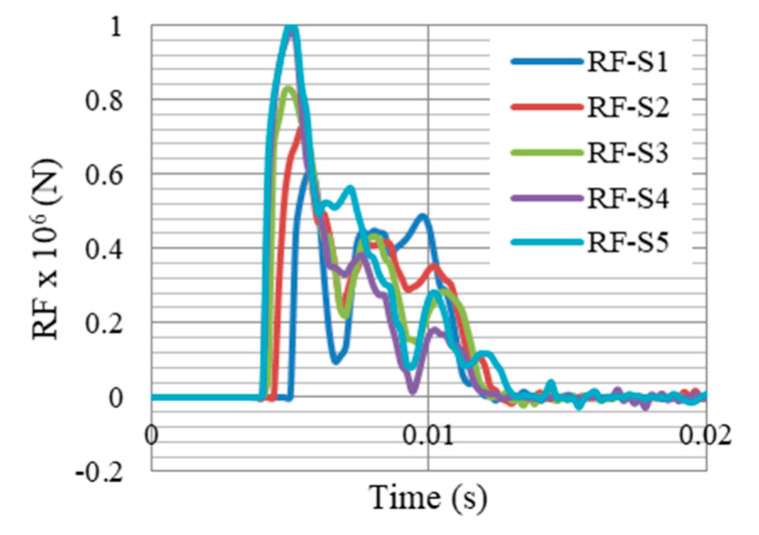
Reaction forces RF at supports S1–S5 without external dampers, after peak reflected overpressure of 6.6 MPa from 100 kg TNT at R = 5 m, Gate G5.

**Figure 23:**
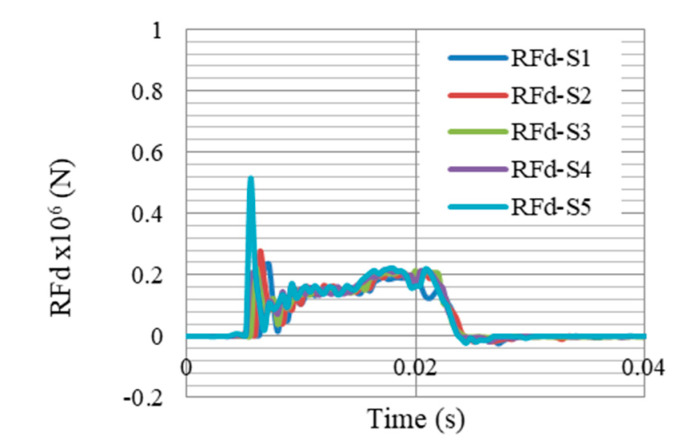
Reaction forces RFd at supports S1–S5 with the auxetic dampers, after peak reflected overpressure of 6.6 MPa from 100 kg TNT at R = 5 m, Gate G5.

The reduction of gate reaction forces was one of the objectives of this paper, which would in return; reduce the required cross section and strength of the whole system supports. Figure 22 and Figure 23 compare the reaction forces at supports S1–S5, without and with the proposed auxetic dampers, respectively. It is clearly shown that peak reaction force (which is at support S5) was dropped from 1 × 10^6^ to 0.51 × 10^6^ N (49% of reduction).

**Figure 24:**
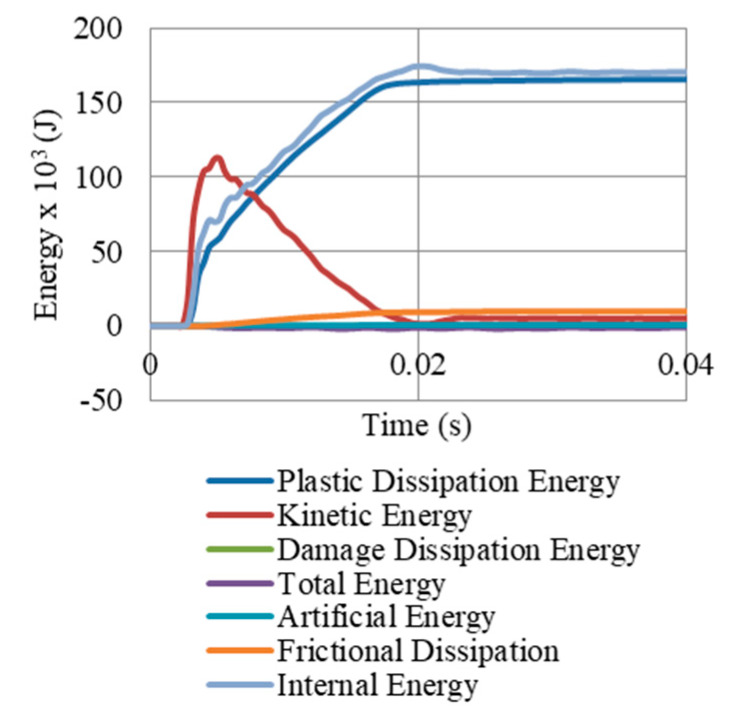
Energy components of the model (shown in Figure 9E), after peak reflected overpressure of 6.6 MPa from 100 kg TNT at R = 5 m, Gate G5.

**Figure 25:**
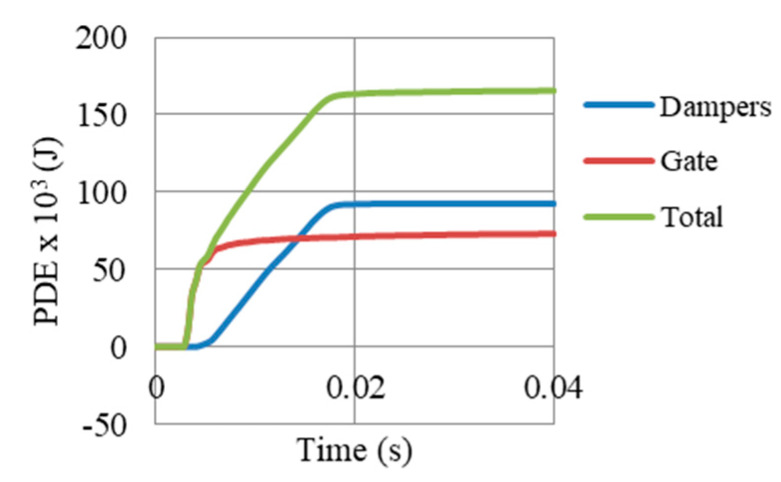
PDE by dampers, gate and the total PDE in the model (shown in Figure 9E), after peak reflected overpressure of 6.6 MPa from 100 kg TNT at R = 5 m, Gate G5.

Figure 24 presents the energy components of the Gate G5 model (shown in Figure 9E), after a peak reflected overpressure of 6.6 MPa. It shows that internal energy in the whole model (174 × 10^3^ J) constitute of major plastic dissipation (164 × 10^3^ J) and minor frictional dissipation (10 × 10^3^ J), with no dissipation due to damage. Based on that successful damping, the kinetic energy is mitigated. It is also important to highlight that the value of artificial energy is near zero, which reflects that the numerical model of the system was accurate to high extent. In addition, Figure 25 shows that 56% of the total PDE in the system was achieved from the UGADs, while 44% from the gate. The additional PDE gained from those light weight auxetic cores justifies the significant reduction in permanent deformations and reaction forces.

**Figure 26:**
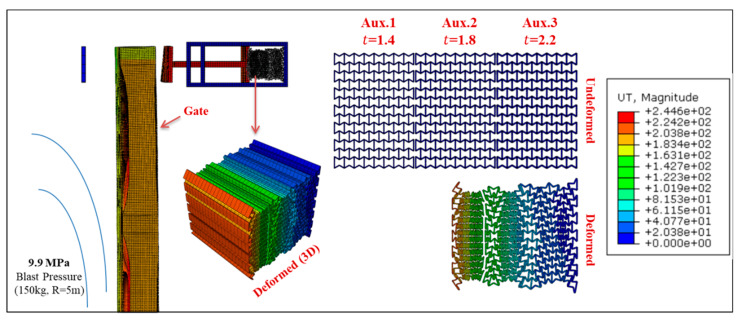
Displacement of Gate G5 and the Auxetic damper after a blast peak reflected overpressure of 9.9 MPa from 150 kg TNT at R = 5 m.

Despite the fact that the peak reflected overpressure taken in this study as target was 6.6 MPa (from 100 kg TNT at R = 5 m), it is worth checking the behaviour of the system beyond that limit. Figure 26 shows the displacement of Gate G5 and the auxetic damper after peak reflected overpressure of 9.9 MPa (from 150 kg TNT at R = 5m), i.e., 1.5 times more than the target. The third auxetic core (Aux.3) was surprisingly able to absorb the additional pressure without a full crash of the gate on to the supports. In addition, the gate maintained its integrity preventing access to the premises. However, the gate exceeded the operability limit with large permanent deformations and aesthetic defects. If such an extreme blast level is expected on site, then it might be needed to use Gate G7.5 instead.

Finally, one may suspect that the piston rod (shown in Figure 8a) would withstand peak RFd of 500,000 N (Figure 23). Therefore, the strength and lateral buckling are checked here. If the cross-sectional dimensions are 35 mm × 35 mm, the peak stress in the rod would be 408 MPa, less than the yield point of the steel material used. In terms of buckling, according to Euler’s formula $P_{cr}=\pi^{2}EI/{\left(KL\right)}^{2}$, the critical load $P_{cr}$ can be calculated, beyond which a column would buckle. The modulus of elasticity E is given as 200 × 10^3^ MPa. Moment of inertia I = $\frac{bh^{3}}{12}=\frac{35^{4}}{12}=125,052$ mm^4^. The effective length factor K is 2 for free-end column, and the unsupported length of the column is 310 mm. Then, critical load $P_{cr}$ is 642 150 N, greater than the applied axial load. In other words, the piston rod would stay in elastic range with no lateral buckling, when subjected to peak reaction forces generated from 100 kg TNT at 5 m. In addition, numerical results showed no local buckling or eventual crippling in the piston rod.

## 9. Conclusions

The structural response of four gates, namely G2.5, G5, G7.5, and G10, were numerically assessed. Site and threat possibilities were described in addition to geometrical and material properties of the structure. Then, the numerical model was validated based on detailed mesh analysis. The analysis looked at five fields, namely reaction forces, maximum plastic strain, permanent deformation, operability, and energy components. The following points outline main conclusions of this research:

- In-plane reaction forces are very small compared to those out-of-plane (direction of blast). Therefore, RFz is the considered component in this paper as it is the prominent one.
- The UGAD dampers may work for G5, G7.5, or G10 in the same efficiency, as the mass shown to have slight effect on RFs (Figure 11).
- G10 was the only gate (without external damping systems) that satisfied operability condition after the blast event, with peak permanent deformation, *d*_frame_ = 4.4 mm.
- With the application of the proposed UGAD, both G7.5 and G5 passed the operability requirement. The frame permanent deformation of G5 decreased from 40.5 to 22 mm, making G5 the lightest-operable option that can withstand the peak reflected overpressure target of 6.6 MPa. In addition, a 49% reduction in peak reaction forces was recorded which can reduce the required cross section and strength of the concrete supports.
- Internal energy in the whole model composed mainly of plastic dissipation, small frictional dissipation, and no dissipation due to damage. Moreover, 56% of the total plastic dissipation energy in the system was achieved from the UGADs, while 44% from the gate. Based on that successful energy dissipation, the kinetic energy was mitigated.

In short, the paper proposes an innovative light-weight blast resistant steel gate supported with the recently introduced UGADs. The additional plastic dissipation energy gained from those sacrificial auxetic cores justifies the significant reduction in permanent deformations and reaction forces of the steel gate. The working mechanism of the gate structure proposed in this research is thought to be suitable for various buildings/door sizes. The outcomes of this research may have a positive impact on other sectors beyond academia, such as industry, economy, and public safety. The author’s interest for future research is the design of a reinforced concrete structure that can support the steel gate and the UGADs. Proposals for this reinforced concrete structure are introduced in [49,50].

## Figures and Tables

**Table 1:** Material parameters for Weldox 460E Steel (adopted from [32]).

Category	Constant	Description	Unit	Value
Elastic Constants	*E*	Modulus of Elasticity	MPa	200 × 10^3^
	*ν*	Poisson’s ratio	-	0.33
Density	*ρ*	Mass density	t/mm^3^	7.85 × 10^−9^
Yield stress and strain hardening	*A*	Yield Strength	MPa	490
	*B*	Ultimate Strength	MPa	807
	*n*	Work-hardening exponent	-	0.73
Strain-rate hardening	$\dot{\varepsilon_{0}}$	Reference Strain rate	S^−1^	5 × 10^−4^
	*C*	Strain rate factor	-	0.0114
Damage evolution	$D_{c}$	Critical Damage	-	0.3
	$p_{d}$	Damage threshold	-	0
Adiabatic heating and temperature softening	$C_{p}$	Specific heat	mm^2^K/S^2^	452 × 10^6^
	*χ*	Taylor Quinney empirical constant/inelastic heat fraction	-	0.9
	*α*	Coefficient of thermal expansion	K^−1^	1.1 × 10^−5^
	$T_{m}$	Melting Temperature	K	1800
	$T_{0}$	Room Temperature	K	293
	*m*	Thermal-softening exponent	-	0.94
	*K*	-	-	0.74
Fracture Strain Constants	$d_{1}$	-	-	0.0705
	$d_{2}$	-	-	1.732
	$d_{3}$	-	-	−0.54
	$d_{4}$	-	-	−0.015
	$d_{5}$	-	-	0

**Table 2:** The average error in percentage (%) of a specific mesh size compared to mesh size 5 mm.

	Mesh = 50 mm	Mesh = 20 mm	Mesh = 10 mm
Plastic dissipation Energy	12.84	2.85	0.84
Peak reaction force	46.79	32.52	0.80

**Table 3:** The 3 auxetic cores of the Uniaxial Graded Auxetic Damper (UGAD), with their geometric and mechanical properties, adopted from [31].

	Aux.1	Aux.2	Aux.3
Shape	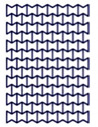	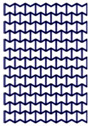	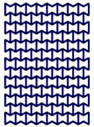
Shared parameters	L = 10 mm, cell angle θ = 60°, Grade AL3 ($\rho_{s}$ = 2.703 × 10^−9^ t/mm^3^), Size = 140 mm × 200 mm × 200 mm, volume of one core V = 5.6 × 10^6^ mm^3^		
t (mm)	1.4	1.8	2.2
*t*/*L*	0.14	0.18	0.22
Mass (ton)	0.00338	0.00434	0.00530
Mass (kg)	3.38	4.34	5.30
Density ρ (t/mm^3^)	6.036 × 10^−10^	7.75 × 10^−10^	9.46 × 10^−10^
Relative Density $\rho^{*}=\rho /\rho_{s}$	0.223	0.287	0.35
Void ratio %	77.7	71.3	65

**Table 4:** Mass of the 4 gates G2.5, G5, G7.5 and G10.

Gate	G2.5	G5	G7.5	G10
Total Mass (ton)	1.10	2.19	3.29	4.38
Mass/Area (kg/m^2^)	81.12	162.23	243.35	324.47

**Table 5:** Plastic strain, permanent deformation and operability for the 4 gates under consideration, subjected to 6.6 MPa peak reflected overpressure from 100 kg TNT at R = 5 m.

		Peak Plastic Strain			Permanent Deformation *d* (mm)			Operable
Gate	*t* (mm)	Frame	Front Plate	Back Plate	Frame	Front Plate	Back Plate	(Yes/No)
G2.5	2.5	0.89	0.82	0.17	551.0	489.0	490.0	No
G5	5	0.29	0.17	0.25	40.5	65.6	40.0	No
G7.5	7.5	0.20	0.13	0.17	28.4	30.0	28.0	No
G10	10	0.02	0.07	0.05	4.4	11.6	10.5	Yes

**Table 6:** Plastic strain, permanent deformation and operability of the gates with the proposed auxetic damper, subjected to 6.6 MPa blast peak reflected overpressure from 100 kg TNT at R = 5 m.

		Peak Plastic Strain			Permanent Deformation *d* (mm)			Operable
Gate	*t* (mm)	Frame	Front Plate	Back Plate	Frame	Front Plate	Back Plate	(Yes/No)
G2.5	2.5	0.93	0.89	0.19	676	613	609	No
G5	5	0.1	0.17	0.156	22	51	24	Yes
G7.5	7.5	0.03	0.16	0.1	4	19	8	Yes
